# Casamino acids facilitate the secretion of recombinant dengue virus serotype-3 envelope domain III in *Pichia pastoris*

**DOI:** 10.1186/s12896-016-0243-3

**Published:** 2016-02-04

**Authors:** Neha Kaushik, Deepak Rohila, Upasana Arora, Rajendra Raut, Urpo Lamminmäki, Navin Khanna, Gaurav Batra

**Affiliations:** Centre for Biodesign and Diagnostics, Translational Health Science and Technology Institute, NCR Biotech Science Cluster, Faridabad, Haryana India; Recombinant Gene Products Group, International Centre for Genetic Engineering and Biotechnology, New Delhi, India; Department of Biochemistry/Biotechnology, University of Turku, Turku, Finland

**Keywords:** Casamino acids, Dengue virus, Envelope domain III, *Pichia Pastoris*, Secretion

## Abstract

**Background:**

Dengue is a viral disease spread to humans by mosquitoes. Notably, there are four serotypes of dengue viruses (DENV) that places ~40 % of the global population at risk of infection. However, lack of a suitable drug or a preventive vaccine exacerbates the matter further. Envelope domain-III (EDIII) antigen of dengue virus (DENV) has garnered much attention as a promising vaccine candidate for dengue, in addition to its use as a diagnostic intermediate. Hence developing a method for efficient production of high quality recombinant EDIII is important for research and industrial purpose.

**Results:**

In this work, a *Pichia pastoris* system was optimized for the secretory over-expression of DENV serotype-3 EDIII under the control of methanol inducible AOX1 promoter. Temperature alone had a significant impact upon the amount of secretory EDIII, with 2.5-fold increase upon reducing the induction temperature from 30 to 20 °C. However surprisingly, supplementation of culture media with Casamino acids (CA), further augmented secretory EDIII titer, with a concomitant drop of intracellular EDIII levels at both temperatures. Though, reduction in intracellular retention of EDIII was more prominent at 20 °C than 30 °C. This suggests that CA supplementation facilitates overexpressing *P. pastoris* cells to secrete more EDIII by reducing the proportion retained intracellularly. Moreover, a bell-shaped correlation was observed between CA concentration and secretory EDIII titer. The maximum EDIII expression level of 187 mg/L was achieved under shake flask conditions with induction at 20 °C in the presence of 1 % CA. The overall increase in EDIII titer was ~9-fold compared to un-optimized conditions. Notably, mouse immune-sera, generated using this purified EDIII antigen, efficiently neutralized the DENV.

**Conclusions:**

The strategy described herein could enable fulfilling the mounting demand for recombinant EDIII as well as lay direction to future studies on secretory expression of recombinant proteins in *P. pastoris* with CA as a media supplement.

**Electronic supplementary material:**

The online version of this article (doi:10.1186/s12896-016-0243-3) contains supplementary material, which is available to authorized users.

## Background

Dengue, a mosquito-borne disease, can be caused by four antigenically distinct serotypes of dengue viruses (DENVs). Recent analysis suggests that DENVs causes as many as 390 million (95 % credible interval 284–528) infections annually [1]. However, there is no vaccine or drug available in the market to fight against this global public health problem. Accumulating evidences indicate the potential of dengue virus (DENV) Envelope Domain III (EDIII) as a possible vaccine candidate, in addition to its usage as a highly specific diagnostic intermediate and a plausible target for neutralizing therapeutic antibodies [2]. EDIII, that spans amino acid (aa) residues 297–400 of the E protein, is an immunoglobulin (Ig)-like domain. It is implicated in the binding of the virion to the host cell surface receptor and contains multiple type and sub-type specific neutralizing epitopes [3]. Identity amongst the EDIIIs of the four DENV serotypes is 60–70 % (Additional file 1: Figure S1), yet these domains are antigenically distinct and can be used for the serotyping of immune response [2, 4]. Because of its unique features, recombinant versions of EDIII have also found extensive utility in basic research, for understanding host-virus interactions, and immune response against natural infection [2, 3].

Numerous expression systems have been reported for the production of recombinant EDIII from different DENV serotypes, however *E. coli* remains the most widely used expression host [4–6]. Soluble expression of EDIII in *E.coli* involves the fusion of protein with a large solubility tag, such as MBP [5]. In the absence of a solubility tag, overexpressed EDIII tends to be insoluble and hence necessitates purification under denaturing conditions, followed by renaturation [4, 6].

The yeast *Pichia pastoris* offers several advantages as a protein production host. It harbors a strong yet tightly regulated alcohol oxidase I (AOX1) promoter that allows for efficient expression of recombinant protein. *P. pastoris* also allows for very high levels of extracellular secretion of recombinant protein into culture media with minimal secretion of endogenous proteins [7].

Use of *P. pastoris* for the secretory expression of DENV serotype-2 EDIII with shake flask, achieving titer of 2.2 mg/L, and bioreactor, achieving titer of 172 mg/L has been reported [8]. Secretion of DENV serotype-1 EDIII in *P. pastoris* has also been shown but without any information on the titer of the secreted protein [9].

Here we report the construction and optimization of a secretory *P. pastoris* clone of DENV serotype-3 EDIII for high-level secretion of EDIII into minimal media. We show for the first time a dosing effect of CA supplementation in culture media on the secretory expression of recombinant protein in *P. pastoris*. Our data clearly suggest that CA supplementation increases the secretory titer of recombinant EDIII by reducing the intracellular retention of recombinant protein.

## Methods

### Reagents

Anti-penta His mAb, Ni-NTA superflow resin and Ni-NTA His-Sorb plates were procured from Qiagen GmbH, Germany. Anti-mouse IgG-H&L-chain-HRP-conjugate was from JIR, USA. Acid washed glass beads (425-600 microns), HRP substrate TMB were from Sigma-Aldrich, USA. Montanide ISA720 was from Seppic Inc., France. BCA protein assay reagent was from Thermo Scientific, Rockford, USA. Vero cell lines and Pan-DENV prM-specific 2H2 mAb were obtained from ATCC, USA. DENV EDIII-specific mAb 24A12 was generated in-house. Alexa Fluor 488 for labeling mAbs was from Life Technologies. N1-Europium chelate was synthesized in-house at University of Turku, Finland. DENV-3 EDIII gene, codon-optimized for *P. pastoris* expression, was obtained from GeneArt AG, Germany. Casamino Acids (Bacto casamino acids: acid-hydrolyzed casein, low sodium chloride and iron concentrations) Cat # 223050 from Becton, Dickinson and company, USA. CA are a mixture of amino acids and some very small peptides (<5 amino acids) obtained from acid hydrolysis of casein.

### Generation of DENV-3 EDIII expressing *P. pastoris* clone

The synthetic codon-optimized *DENV-3 EDIII* gene (Additional file 2: Figure S2) was cloned under the control of *AOX1* promoter of pPICZαA vectors (Life Technologies) and integrated into the genome of *P. pastoris* Mut^S^ strain KM71H. Keeping future requirements in mind, Mut^s^ (methanol utilizing slow) was preferentially selected over a Mut^+^ (methanol utilizing plus) strain owing to the ease of handling of the former during large-scale production. In contrast to Mut^+^, methanol accumulation does not hyper-accelerate the growth of Mut^s^, and hence enables better control over heat and oxygen demand during large-scale production [10].

Transformants obtained through zeocin selection, were screened for EDIII expression. Test tube cultures of 30 EDIII transformants and 1 negative control (KM71H containing empty pPICZαA) were set up in 3 ml YPD and incubated for 72 h at 30 °C with shaking at 300 rpm, ensuing equal confluence. From these pre-cultures, 50 μl aliquots were used to inoculate 8 ml buffered glycerol-complex media (BMGY) [1 % yeast extract, 2 % peptone, 100 mM Potassium phosphate, pH 5.8; 1.34 % YNB; 4x10^−5^% Biotin; 1 % Glycerol] in duplicate tubes. Cultures were allowed to shake at 300 rpm, at 30 °C for 30 h (OD_600_ = ~25). Cells were pelleted by low speed centrifugation (1500× *g*) and re-suspended in 3 ml buffered methanol-complex media (BMMY) [1 % yeast extract, 2 % peptone, 100 mM Potassium phosphate, pH 5.8; 1.34 % YNB; 4×10^−5^% Biotin; 2 % Methanol]. Inductions were maintained for 3 days at 30 °C. During this time, 2 % (v/v) methanol was added at 24 h intervals. After 3 days of induction, cultures were centrifuged and supernatant were used for immunoassay for the determination of EDIII secretion.

### Culture condition optimization

The best secretory-clone was used for optimization experiments in shake-flask setting. The growth phase was performed in buffered minimal glycerol (BMG) [100 mM Potassium phosphate, pH 5.8; 1.34 % YNB; 4×10^−5^% Biotin; 1 % Glycerol] and induction phase in buffered minimal methanol (BMM) [100 mM Potassium phosphate, pH 5.8; 1.34 % YNB; 4×10^−5^% Biotin; 2 % Methanol]. To optimize culture conditions to increase the secretion level of EDIII protein, effect of induction temperature (20 °C and 30 °C) and CA supplementation, at different concentrations (0.25, 0.5, 1.0, 1.5 and 2 % w/v), in the BMM media was investigated. A starter culture was set up by inoculating 50 ml YPD medium with the *P. pastoris* EDIII clone #27 glycerol stock and incubated for ~20 h at 30 °C with shaking at 270 rpm. This starter was used to inoculate 1500 ml of BMG to a starting OD_600_ = 0.08 and divided equally into three 2 L baffled flasks (500 ml each). Culture was allowed to grow at 30 °C with shaking, at 270 rpm, for ~24 h (OD_600_ = ~30). Cells were harvested by centrifuging at 1500×*g* for 5 min at RT, and washed with sterile double-distilled water. Cells were resuspended in water to an OD_600_ = 120 and 25 ml of this suspension was added into twelve 250 ml baffled flasks prefilled with 25 ml of 2× BMM media and appropriate 2× [CA]. The resulting cultures therefore had a starting, OD_600_ of 60 in 1× BMM containing different concentration of CA (0, 0.25, 0.5, 1.0, 1.5 and 2 % w/v). The initial OD at the start of induction was reconfirmed at 60 (+/-0.5). Inductions were allowed to proceed for 6 days at 20 °C (6 flasks with 0, 0.25, 0.5, 1.0, 1.5 and 2 % w/v CA) and at 30 °C (6 flasks with 0, 0.25, 0.5, 1.0, 1.5 and 2 % w/v CA). Daily methanol feed of 2 % (v/v) was added in each flask to maintain the induction. In all the above-mentioned cases, culture samples were withdrawn at 24 h intervals, centrifuged, and the clarified supernatants were collected and further used for SDS-PAGE, western blot and immunoassay. Cell pellets were used for extraction of total intracellular proteins.

### Total intracellular protein extraction

To extract total intracellular protein, a buffer containing strong denaturant was used. Cell pellet of 1 ml culture was re-suspended in 200 μl of lysis buffer [6 M Guanidine-HCl, 50 mM Dithiothreitol, 50 mM phosphate buffer pH 7.0]. An equal volume of glass beads were added and cells were allowed to lyse overnight on plate shaker (Eppendorf Mix mate) at 1500 rpm, 25 °C. The lysed cells were centrifuged and the supernatant containing total protein was collected.

### Analytical methods

EDIII levels were determined using customized immunoassay as reported earlier [8] except the use of Europium labeled anti-EDIII-MAb 24A12 instead of anti-EDIII-MAb 3H5 coupled with secondary anti-mouse-HRP. Each sample was assayed in duplicate wells. A reference curve (R^2^ = 0.98), generated in parallel using serially diluted (2-fold) pure DENV-3 EDIII protein (purified as mentioned below) of known concentration (measured using absorbance at 280 nm), was used to determine the EDIII levels in crude samples. Western blot analysis was done using anti-EDIII 24A12 mAb & goat anti-mouse HRP as primary & secondary antibody respectively. For the clone screening immunoassay, anti-EDIII 24A12 mAb and goat anti-mouse HRP were used as primary & secondary antibody respectively.

### Large-scale expression and purification

For the large-scale cultivation and expression of EDIII, essentially the same protocol was followed as stated previously [8], except that BMM media was supplemented with 1 % CA and induction was performed at 20 °C. The Ni-NTA based protein purification protocol were performed as previously reported [8].

### Mice immunization and seroanalysis

Animal experiments were reviewed and approved by the International Centre for Genetic Engineering and Biotechnology institutional animal ethics committee and adhered to the guidelines of the Government of India. A group of five BALB/c mice (4–6 weeks old) was immunized intraperitoneally on days 0, 21 and 42 with 20 μg purified EDIII formulated in Montanide ISA 720 adjuvant. Mock immunizations were performed in parallel wherein PBS replaced the antigen solution in the control group. The neutralizing capacity of immune sera were measured using Flow-cytometry based neutralization test (FNT) as described earlier [11]. In the FNT assay, antibodies capable of neutralizing the infectivity of DENV-3 (WHO reference strain CH53489) were measured. Briefly, Vero cells were infected with DENV-3 strain CH-53489 incubated with pooled serum at various dilutions, and the % of cells infected with the virus were evaluated after 24 h by staining them with Alexa 488 labeled 2H2 mAb and reading it on flow cytometer. The serum dilution, which resulted in 50 % reduction in the % of DENV-infected cells in comparison to the control group, has been reported as FNT_50._ FNT assay for test and mock pooled samples were performed in duplicate wells.

No studies were carried out on or with materials derived from human subjects.

## Results and discussion

Synthetic DENV-3 EDIII gene, cloned in-frame with the *S. cerevisiae* α-mating factor secretory signal under the control of methanol inducible AOX1 promoter, was integrated into the genome of *P. pastoris* Mut^S^ strain using zeocin selection strategy.

Total 30 colonies were screened in test tube setting using customized immunoassay. One clone, #27, which secreted the maximal level of EDIII in the culture supernatant (Additional file 3: Figure S3), was used for all further experiments.

This study assesses the effect of low temperature induction and CA supplementation on the secretory titers of DENV-3 EDIII in shake-flask settings. Optimization experiments were performed in minimal media instead of complex media (containing yeast extract and peptones) owing to the ease in purification of secreted recombinant protein from minimal media.

**Fig. 1:**
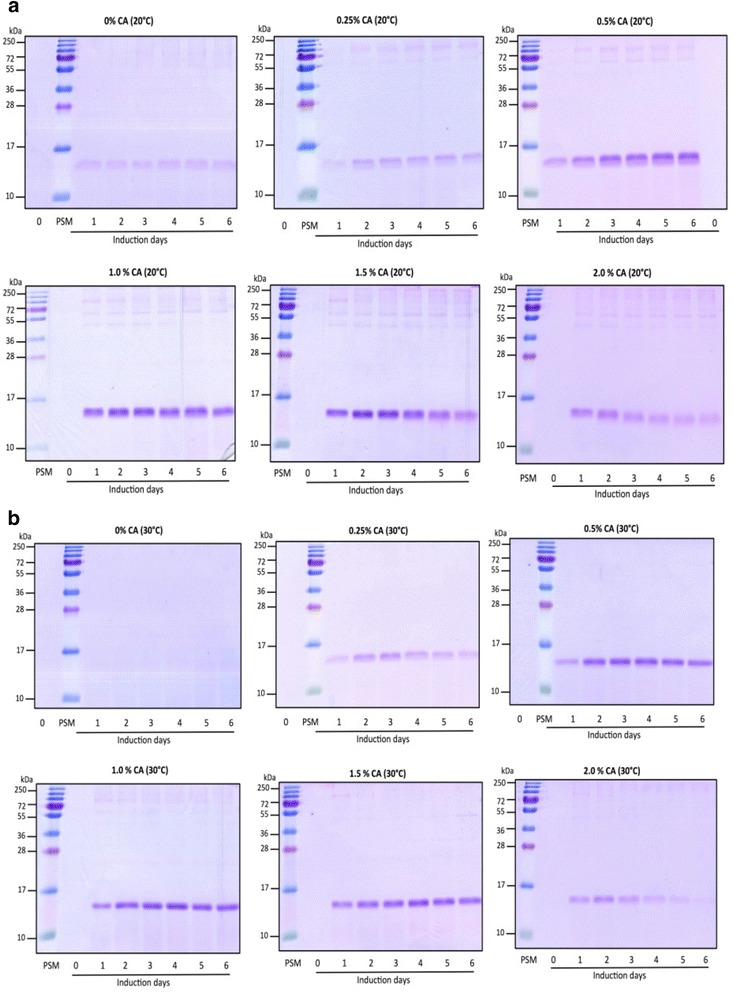
Coomassie stained SDS-PAGE showing secreted EDIII. Presence of secretory EDIII was determined in the supernatant of cultures induced in the absence (CA 0 %) or presence of different concentrations of casamino acids (CA 0.25 % to 2.0 % w/v) supplemented BMM media from day 0 to day 6 at 20 °C (**a**) and 30 °C (**b**). For both panels, similar trends were observed in two independent induction experiments (Additional file 9)

**Fig. 2:**
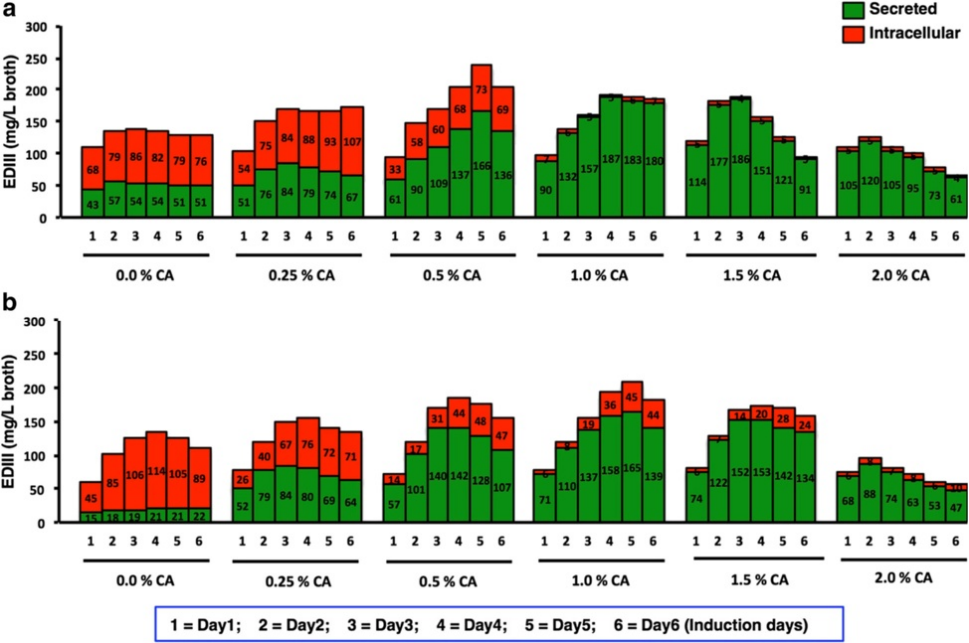
Effect of induction temperature and casamino acids supplementation on the localization of expressed EDIII. Expression was tested at six CA concentrations (0, 0.25, 0.5, 1.0, 1.5, 2.0 % w/v) for 6 days at 20 °C (**a**) and 30 °C (**b**). EDIII, viz. secreted (*green*) and intracellular (*red*) levels were determined using immunoassay and value inside each bar represents EDIII titers in mg/L broth. Similar patterns were observed from culture supernatant (secretory EDIII) and cell mass (intracellular EDIII) from two independent inductions

**Fig. 3:**
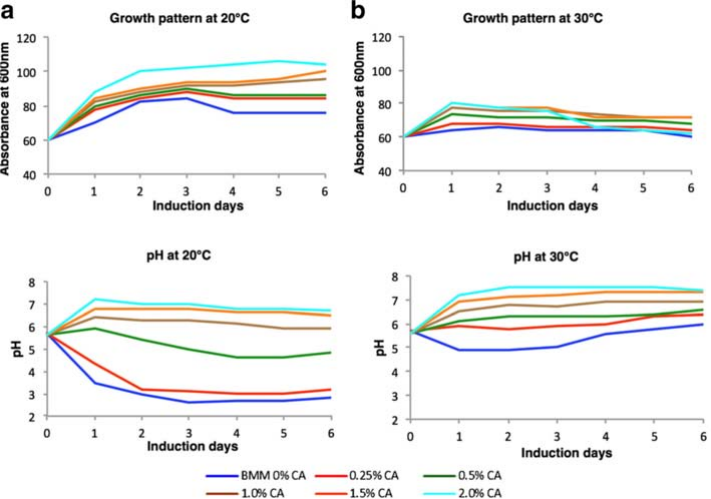
Effect of temperature and CA supplementation on cell growth and culture pH. Cultures induced at 20 °C (**a**) and 30 °C (**b**) were observed for variation in growth profile (*top*) and pH (*bottom*) over time (days). Growth profile was determined by measuring the cell density for each time point at 600 nm. Cells were harvested and pH was measured for the retrieved cell-free media. Tables containing absolute OD_600_ and pH values are also provided as additional information (Additional file 8: Table S8). Similar trends were observed in two independent induction experiments (Additional file 9)

The effects of low temperature induction upon protein expression and secretion have been well studied. Higher secretory titers of recombinant protein at lower temperature has been associated with lower cell death [12] and slower translation ensuring reduced ER stress [13, 14]. In concurrence, induction at 20 °C had a significant impact upon secreted EDIII titer in our study (Figs. 1 and 2). In contrast to 30 °C, a distinct band corresponding to EDIII (~14 kDa) was observed when the culture was induced at 20 °C (Fig. 1). The total secreted EDIII in culture supernatant was estimated at ~54 mg/L broth for 20 °C as against ~21 mg/L broth at 30 °C (Fig. 2). Notably a cognate 1.3 fold higher cell mass was also observed at 20 °C compared to 30 °C (Fig. 3), which is consistent with earlier report [12]. However, an increase in accumulation of secreted EDIII was not observed over time.

There are several reports suggesting a positive effect of media supplementation with CA on the titer of secretory proteins in yeast system [15–21]. CA supplementation has been associated with accumulation of secretory protein over a longer induction period [15]. Additionally, some of these studies have also tried to link the beneficial effect of CA with reduction in proteolytic activity in culture media [15, 18, 20]. However hitherto, no report has provided a concrete explanation for the positive effect(s) of CA.

Further, CA is also an alternative carbon and nitrogen source for cell growth [7]. Moreover, presence of CA in culture media doesn’t interfere in the protein purification methods unlike yeast extract and peptones [22]. Hence to enhance EDIII accumulation over time, we decided to attempt CA supplementation. Every report until now, barring one by Toonkool et al [15] (where two concentrations of CA in the media were evaluated), has used a single concentration of CA [16–21, 23]. Hence, it was worthwhile to evaluate the effect of CA at varying concentrations. Thus, we studied the dosing effect of CA at five different concentrations (0.25–2 % w/v). Induction at 20 °C in the presence of CA resulted in enhanced EDIII secretion at all concentrations (Figs. 1a and 2a). Notably, EDIII secretory titers varied significantly between CA concentrations, with maximum EDIII accumulation (187 mg/L broth) on the 4^th^ day in the culture supplemented with 1 % CA (Figs. 1a and 2a). This was ~3.5 times higher than the cognate condition without CA. Interestingly a enhancement, was also observed for CA supplementation at 30 °C (Figs. 1b and 2b). Surprisingly a ~7.8-fold increase in EDIII secretory titer was observed at 30 °C upon CA addition, bridging the titer gap observed earlier between 20 °C and 30 °C induction in the absence of CA. Moreover, a correlation plot of CA concentration and titer of secretory EDIII was found to be bell-shaped at both temperatures (Additional file 4: Figure S4). This suggests, that unless otherwise optimized, using an arbitrary supplementation of CA will invariably have suboptimal effect upon secretory expression of recombinant protein (Figs. 1 and 2). Notably reports suggesting negative effect of CA supplementation on the accumulation of recombinant secretory protein [23], might be due to the use of non-optimal CA concentration for study.

**Fig. 4:**
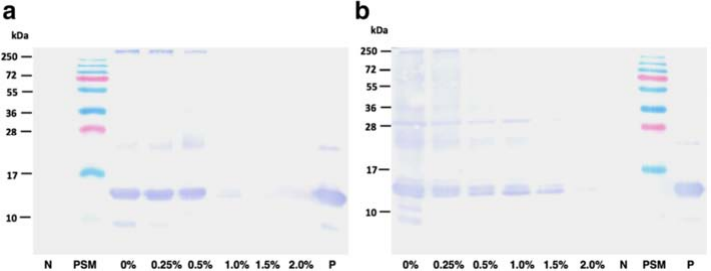
Determination of intracellularly retained EDIII. Western blot of induced *P. pastoris* cell lysate [day 4 harvest with different CA concentration 0.0 to 2.0 % (w/v) supplemented BMM media] to determine intracellular EDIII protein at two induction temperatures. **a** 20 °C induced cell lysate showing presence of ~14 kDa EDIII protein band upto 0.5 % CA (w/v) whereas, **b** 30 °C induced showing gradual decrease in intracellular EDIII protein from 0 to 1.5 % CA (w/v). Anti-EDIII 24A12 mAb & goat anti-mouse HRP were used as primary & secondary antibody respectively. N represents negative control (KM71H cells transformed with pPICZαA) and P represents positive control (purified EDIII). Similar trends were observed with the cell mass (intracellular EDIII) obtained from two independent inductions (Additional file 9)

A higher growth rate (~1.3 fold) (Fig. 3a and b top) alone in the presence of CA however, cannot rationalize the observed unprecedented increase in EDIII titer. We could also observe a buffering effect of CA on culture pH when induced at 20 °C (Fig. 3a, Bottom). Such an effect of CA has also been reported by others [15, 16]. But, this might not be a decisive factor for enhanced EDIII secretion, since the pH of the culture was also stable when induced at 30 °C in the absence of CA (Fig. 3b, Bottom). The reduction of pH at 20 °C in the absence of CA might be associated with the higher growth at 20 °C compared to 30 °C (Fig. 3). We have also observed higher growth and similar reduction in pH at 20 °C in BMM for KM71H cells harboring gene-less pPICZαA cassette (Additional file 5: Figure S5). CA has been associated with reduction of proteolytic activity in culture broth [7, 15, 18, 20]. However, this could only be a minor factor in enhanced EDIII accumulation, as we did not observe any significant proteolysis of EDIII in the absence of CA (Additional file 6: Figure S6). Enhancement in secretory EDIII titer was visible from Day 1 post induction in the presence of CA, negating a mere stabilizing role of CA. In agreement, a significant decline in retained (intracellular) EDIII levels was observed upon CA supplementation in a dose-dependent manner (Fig. 2). Western blot analysis of normalized total cell lysates corroborated with this finding as EDIII was barely detected in cells induced in the presence of CA at both 20 °C (>0.5 % CA) and 30 °C (2 % CA) (Fig. 4). The ratio of secreted/intracellular EDIII on the 4^th^ day of induction was measured at 0.62 (0 % CA, 20 °C), 37.4 (1 % CA, 20 °C), 0.18 (0 % CA, 30 °C) and 4.38 (1 % CA, 30 °C). These results clearly suggest a CA-dependent increase in EDIII secretion, however the reduction in intracellular retention of EDIII is less prominent at 30 °C compared to 20 °C.

Intracellular accumulation of protein, intended for secretion, is well described and congestion of endoplasmic reticulum (ER) folding and secretion capacity during protein trafficking is the most common rate-limiting factor [7, 13, 24]. However interestingly, intracellularly retained EDIII (without CA or at low CA concentrations) was essentially found to be in a matured form (processed α-prepro leader sequence) especially at 20 °C (Fig. 4a), indicating that the protein was already processed at the Golgi apparatus (pro part of signal sequence cleaved by Golgi resident Kex2 protease). This indicates that ER congestion is not the main factor for intracellular EDIII retention in the absence of CA. It is possible that CA might be modulating some other non-ER checkpoints. Moreover, we cannot exclude the possibility of a cumulative effect of multiple yet unknown mechanisms modulated by CA supplementation.

Multiple strategies have been described to increase the titer of secretory protein including the use of different signal sequences, overexpression of secretion and folding aid(s), manipulation in cell-wall biogenesis and different cultivation techniques [7, 24]. However, no report associating an ameliorating effect of CA supplementation on active secretion of recombinant protein in *P. pastoris* exists. Though, CA supplementation has been reported to increase the accumulation of secreted recombinant protein in culture supernatant in yeast, it have often been linked to reduction in proteolytic activity in culture media [7, 15, 18, 20]. Hence, our observation of a CA-dependent increase in secretion of recombinant protein, by reduction in intracellular retention of recombinant protein, makes this report very unique. Also, the observed bell-shaped correlation between CA concentration and secretory EDIII titer (Additional file 4: Figure S4) indicate that using the right concentration of CA for supplementation is key for optimal secretion of recombinant protein. However, deciphering a precise mechanism for the effect will need further experiments.

**Fig. 5:**
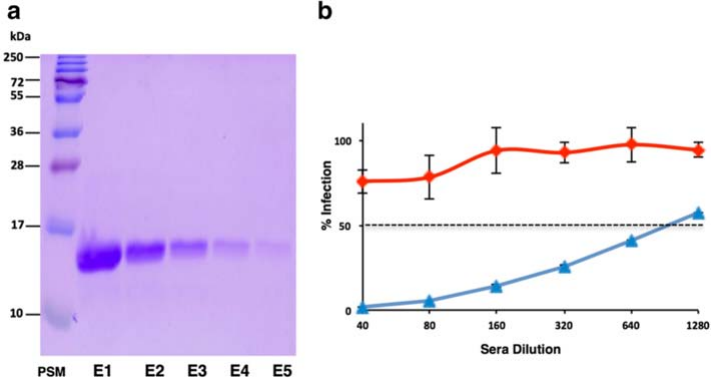
Evaluation of Immunogenicity of purified recombinant EDIII. **a** Coomassie stained 15 % SDS-PAGE showing purified recombinant EDIII as a ∼ 14 kDa band in five separate elution fractions (E1 to E5) after Ni-NTA chromatography. **b** Determination of anti-DENV serotype-3 neutralizing antibody titers using flow-cytometry based neutralization test. Mouse anti-EDIII pooled antisera (*blue*) and mock pooled antisera (*red*) were tested at various dilutions (*X-axis*) and percent viral infection (*Y-axis*) was calculated. The dotted line represents 50 % infection. The error bars represent standard deviation of two technical replicate

Yet another feature of the devised strategy is the titer of recombinant protein obtained (187 mg/L broth). This is the highest reported titer for soluble EDIII, of any flavivirus, without fusion with solubility enhancing tag. A ~9 fold increase in titer was achieved by supplementing with 1 % CA with reduced induction temperature. Since *P. pastoris* secretes very low amount of host-proteins, secretion of EDIII into minimal media can be considered as the first step of purification as can be seen from the Fig. 1. The secretory EDIII was purified to apparent homogeneity from culture induced at 20 °C in the presence of 1 % CA in single step using Ni-affinity matrix (Fig. 5a). To determine the functionality of purified EDIII in terms of its immunogenicity and ability to elicit virus-neutralizing antibodies, mice immunizations were performed, which resulted in very high ELISA titers of anti-EDIII antibodies (Additional file 7: Figure S7). To determine the potential of anti-EDIII immune sera to block the virus infectivity, a flow cytometry based neutralization assay was performed using Vero cells [11]. This assay revealed that the purified recombinant EDIII elicits virus-blocking antibodies in mice with very high neutralizing titers against DENV-3 with FNT_50_ value of 986 (Fig. 5b). This suggests that the devised *P. pastoris* based over-expression strategy using CA as a media supplement is an efficient system for expressing recombinant EDIII. Moreover, given the significance of EDIII, the method described herein will be of immense value to small diagnostic companies and research laboratories that are not equipped with bioreactor facility for the production of recombinant EDIII. The reported titer of >150 mg/L suffices ~1 million diagnostic tests (~100–150 ng /test) from 1 L of production volume and thus can circumvent the need for repeated culturing and purchase.

## Conclusions

In this study we have devised an efficient strategy for enhanced secretory expression of DENV-3 EDIII in *P. pastoris*. In this process we establish a novel augmenting effect of CA upon EDIII secretion in a temperature independent manner. We also provide evidence supporting the need to decipher the correct concentration of CA for supplementation for optimal secretory expression of functional recombinant protein. To best of our knowledge, the titer obtained is the highest reported titer for soluble EDIII expression without fusion with solubility enhancing tag. The described method can find extensive usage for the production of high quality recombinant secretory proteins in *P. pastoris* in the future.

## Additional files

Additional file 1: Figure S1. Multiple sequence alignment of EDIII protein from DENV serotypes. Completely conserved residues are highlighted in red background whereas partially conserved are boxed. Secondary structure assignments have been made using EsPript3.0 [25] based on PDB ID:3IRC. (PPTX 512 kb) Additional file 2: Figure S2.
*P. pastoris* optimized synthetic DENV 3 EDIII gene. DNA sequence (top) and encoded amino acid sequence (bottom). (PPTX 897 kb) Additional file 3: Figure S3. Identification of the best EDIII-expressing *P. pastoris* clones. Levels of EDIII antigen in induced culture supernatants of several clones were estimated by Immunoassay. The X- and Y-axis indicate the clone numbers and the corresponding EDIII antigen levels in terms of absorbance at 450 nm, respectively. Error bars represent standard deviation of two biological replicate. Anti-EDIII 24A12 mAb & goat anti-mouse HRP were used as primary & secondary antibody respectively. (PPTX 736 kb) Additional file 4: Figure S4. Effect of CA supplementation on the secretory titer of recombinant EDIII. Total secretory EDIII titer (mg/L) for cultures were analyzed by plotting against the concentration of CA (%) used for induction at 20 °C (A) and 30 °C (B). A bell-shaped correlation was observed for CA concentration with the secretory titer of recombinant EDIII, advocating the importance of optimal CA concentration during induction. Error bars represent the standard deviation calculated from two technical replicate. (PPTX 1162 kb) Additional file 5: Figure S5. Effect of induction temperature and CA supplementation on cell growth and culture pH (KM71H harboring empty vector). Cultures of *P. pastoris* KM71H, harboring empty pPICZαA expression cassette, induced at 20 °C (A) and 30 °C (B) were observed for variation in growth profile (top) and pH (bottom) over time (days). Growth profile was determined by measuring the cell density for each time point at 600 nm. Cells were harvested and pH was measured for the retrieved cell-free media. (PPTX 2109 kb) Additional file 6: Figure S6. Evaluating the proteolytic susceptibility of secreted EDIII in culture supernatant. Proteolytic degradation for secreted EDIII was monitored in cell-free culture supernatants to negate the balance between EDIII secretion and degradation. Culture supernatants from *P. pastoris* expressing EDIII in the absence of CA, were obtained after 6 days of induction at 20° and 30 °C. Retrieved supernatants were further incubated at their respective induction temperatures and samples were collected at 0, 28 and 93 h interval from both. Samples were thereafter separated on a denaturing polyacrylamide gel and visualized by Coomassie stain. No visible degradation was observed indicating negligible proteolysis of secreted EDIII. Lane 1: Prestained protein marker; Lane 2–4: 20 °C; Lane 5–7: 30 °C; Lane 2 & 5: 0 h; Lane 3 & 6: 28 h and Lane 4 & 7: 93 h. (PPTX 567 kb) Additional file 7: Figure S7. Evaluation of antibodies elicited by recombinant DENV-3 EDIII using ELISA. Pooled sera from EDIII immunized (solid curve) and mock (dashed curve) BALB/c mice were tested by indirect ELISA using purified EDIII as coating antigen. Goat anti-mouse HRP was used as secondary antibody. Error bars represent the standard deviation calculated from two technical replicate. (PPTX 466 kb) Additional file 8: Table S8. Absolute values (OD_600_ and pH) obtained during induction phase. (DOCX 112 kb) Additional file 9:
**Data from another set of independent induction experiment.** (PDF 1054 kb)

## Abbreviations

AOX1: alcohol oxidase 1
CA: casamino acids
DENV: dengue virus
EDIII: envelope domain III
