# Supplementary figures and images for: Casamino acids facilitate the secretion of recombinant dengue virus serotype-3 envelope domain III in Pichia pastoris
Source: BMC Biotechnol. 2016 Feb 4;16:12. doi: 10.1186/s12896-016-0243-3 (PMC4743106; doi:10.1186/s12896-016-0243-3)

## Slide 1
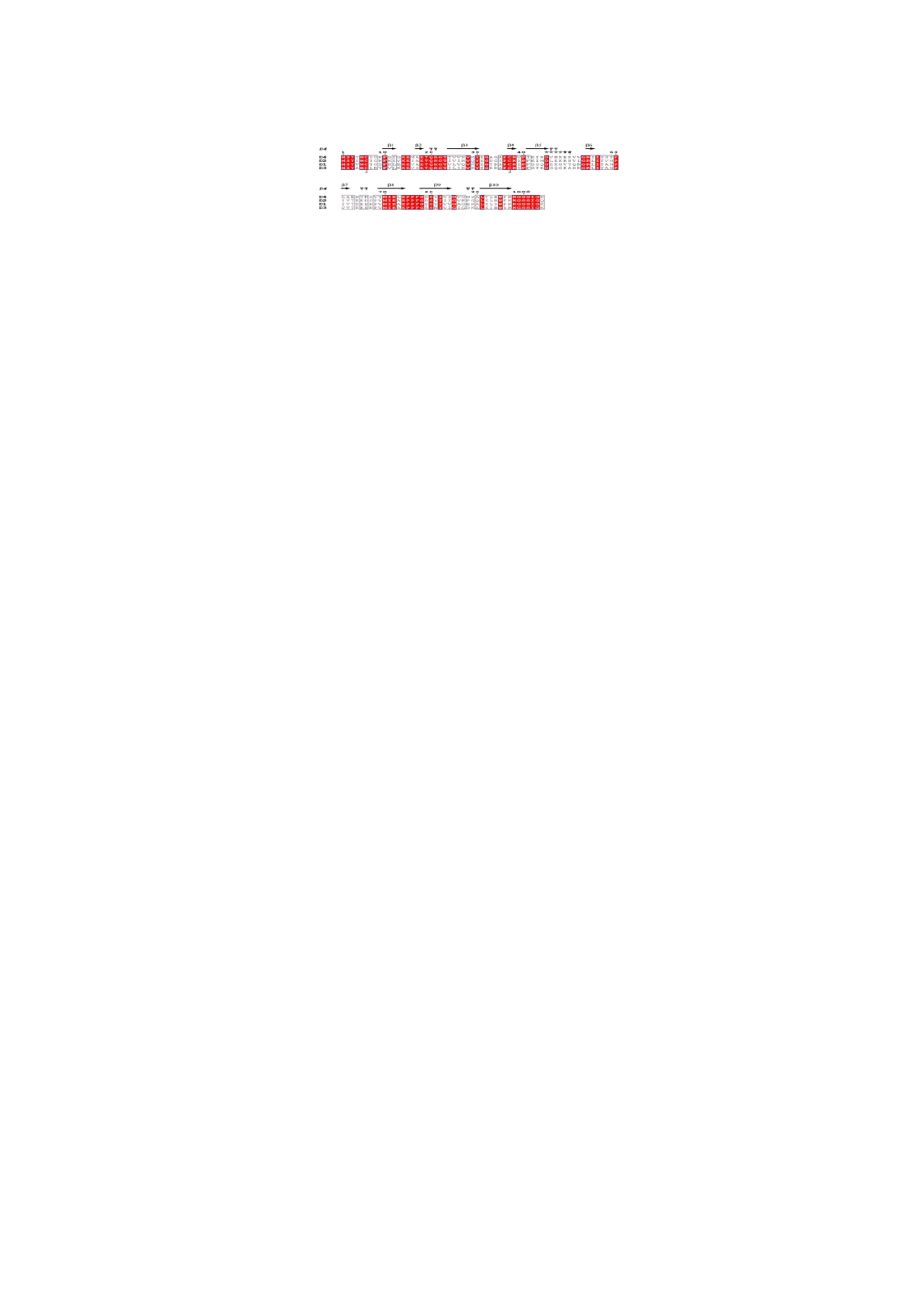

Supplement: Additional file 1: Figure S1. — Multiple sequence alignment of EDIII protein from DENV serotypes. Completely conserved residues are highlighted in red background whereas partially conserved are boxed. Secondary structure assignments have been made using EsPript3.0 [25] based on PDB ID:3IRC. (PPTX 512 kb) [file 12896_2016_243_MOESM1_ESM.pptx]

## Slide 1
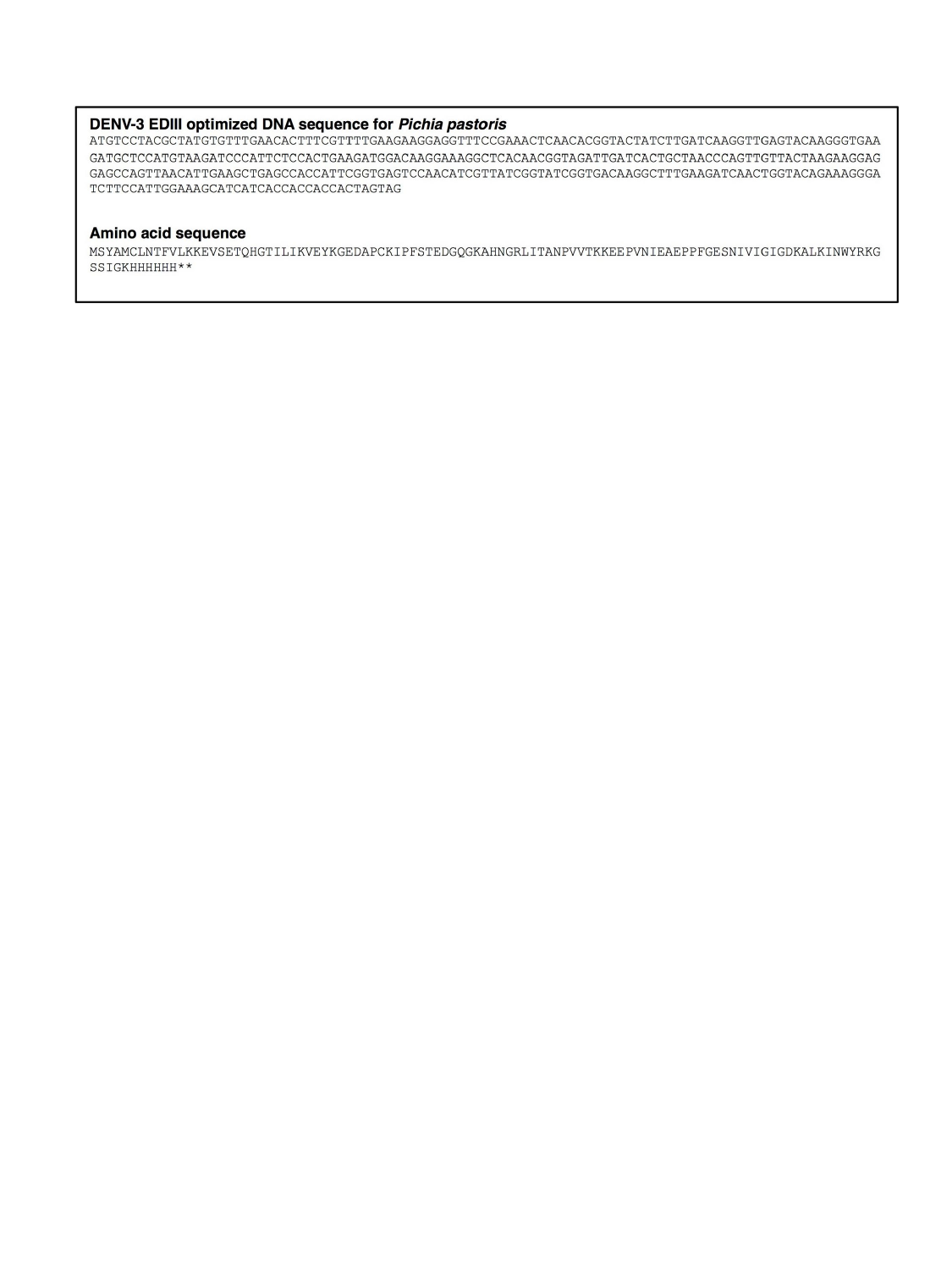

Supplement: Additional file 2: Figure S2. — P. pastoris optimized synthetic DENV 3 EDIII gene. DNA sequence (top) and encoded amino acid sequence (bottom). (PPTX 897 kb) [file 12896_2016_243_MOESM2_ESM.pptx]

## Slide 1
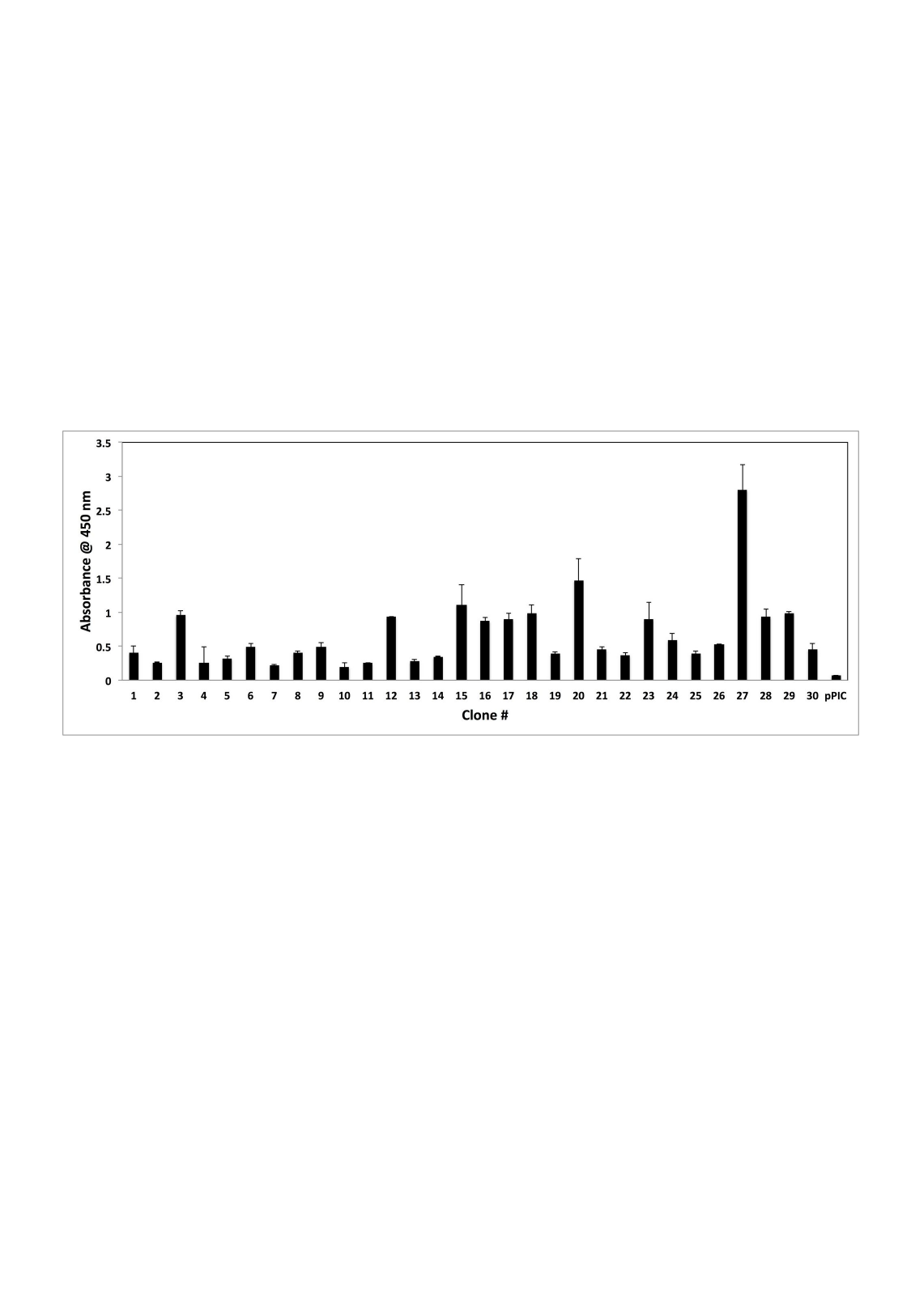

Supplement: Additional file 3: Figure S3. — Identification of the best EDIII-expressing P. pastoris clones. Levels of EDIII antigen in induced culture supernatants of several clones were estimated by Immunoassay. The X- and Y-axis indicate the clone numbers and the corresponding EDIII antigen levels in terms of absorbance at 450 nm, respectively. Error bars represent standard deviation of two biological replicate. Anti-EDIII 24A12 mAb & goat anti-mouse HRP were used as primary & secondary antibody respectively. (PPTX 736 kb) [file 12896_2016_243_MOESM3_ESM.pptx]

## Slide 1
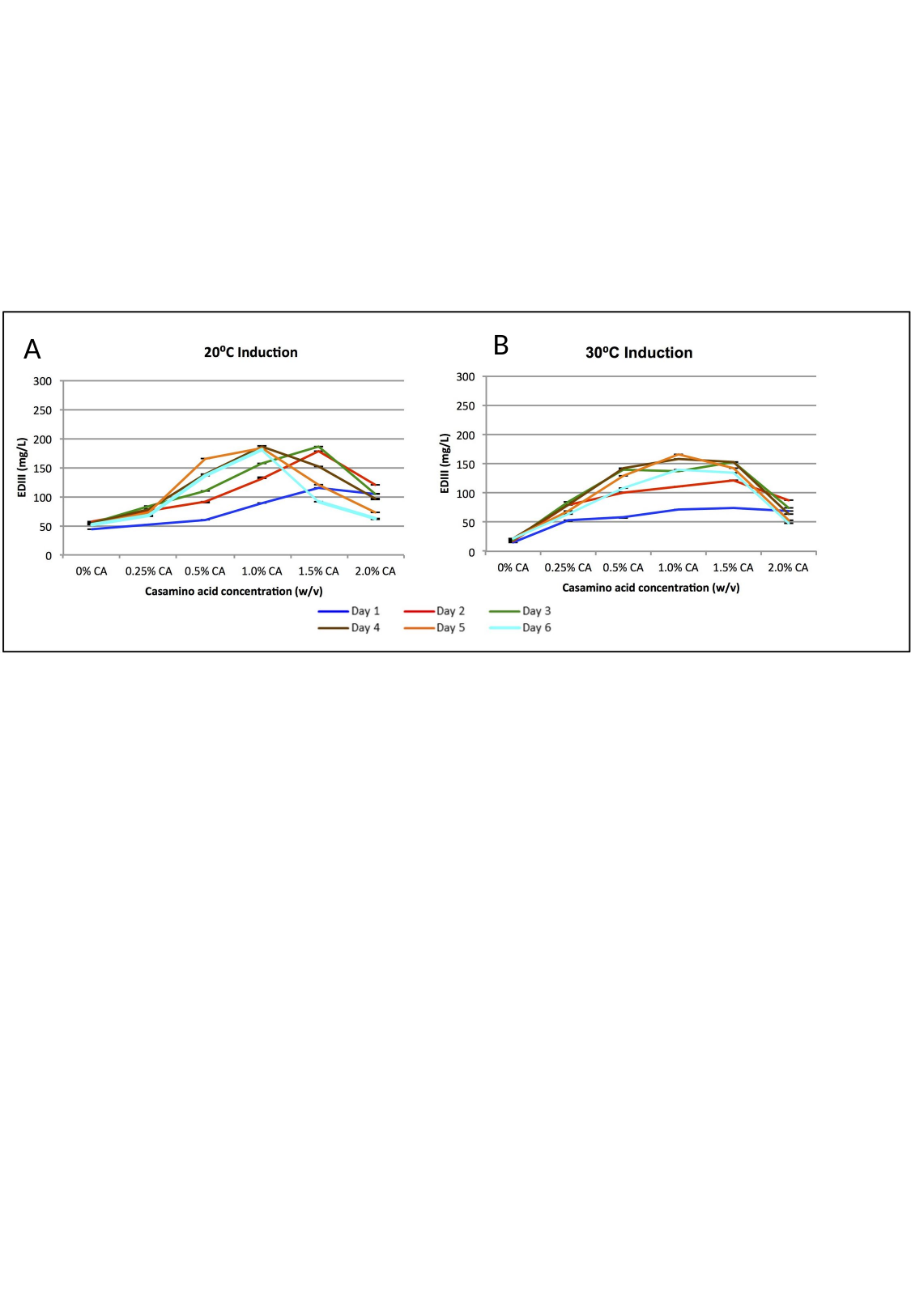

B
A

Supplement: Additional file 4: Figure S4. — Effect of CA supplementation on the secretory titer of recombinant EDIII. Total secretory EDIII titer (mg/L) for cultures were analyzed by plotting against the concentration of CA (%) used for induction at 20 °C (A) and 30 °C (B). A bell-shaped correlation was observed for CA concentration with the secretory titer of recombinant EDIII, advocating the importance of optimal CA concentration during induction. Error bars represent the standard deviation calculated from two technical replicate. (PPTX 1162 kb) [file 12896_2016_243_MOESM4_ESM.pptx]

## Slide 1
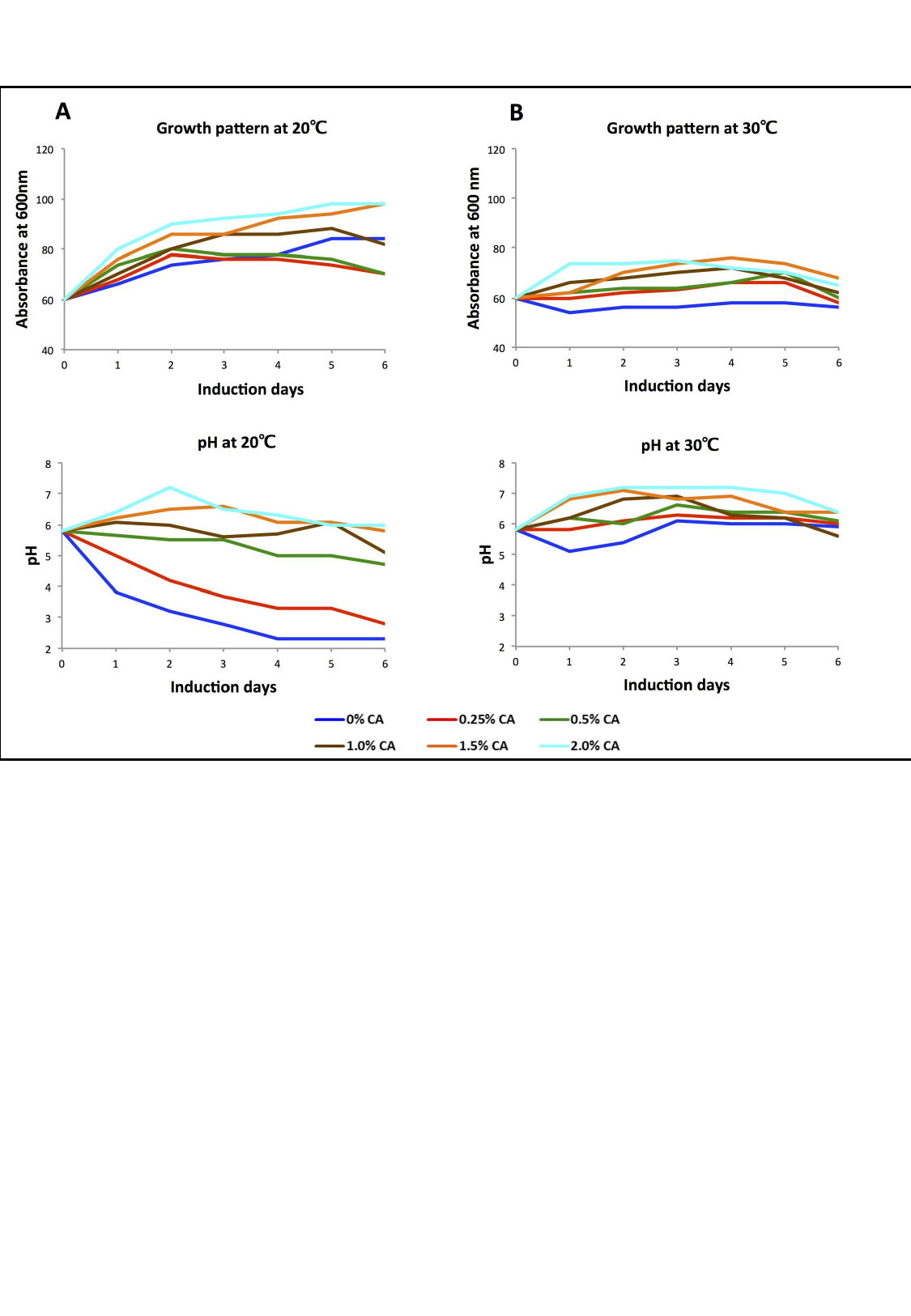

Supplement: Additional file 5: Figure S5. — Effect of induction temperature and CA supplementation on cell growth and culture pH (KM71H harboring empty vector). Cultures of P. pastoris KM71H, harboring empty pPICZαA expression cassette, induced at 20 °C (A) and 30 °C (B) were observed for variation in growth profile (top) and pH (bottom) over time (days). Growth profile was determined by measuring the cell density for each time point at 600 nm. Cells were harvested and pH was measured for the retrieved cell-free media. (PPTX 2109 kb) [file 12896_2016_243_MOESM5_ESM.pptx]

## Slide 1
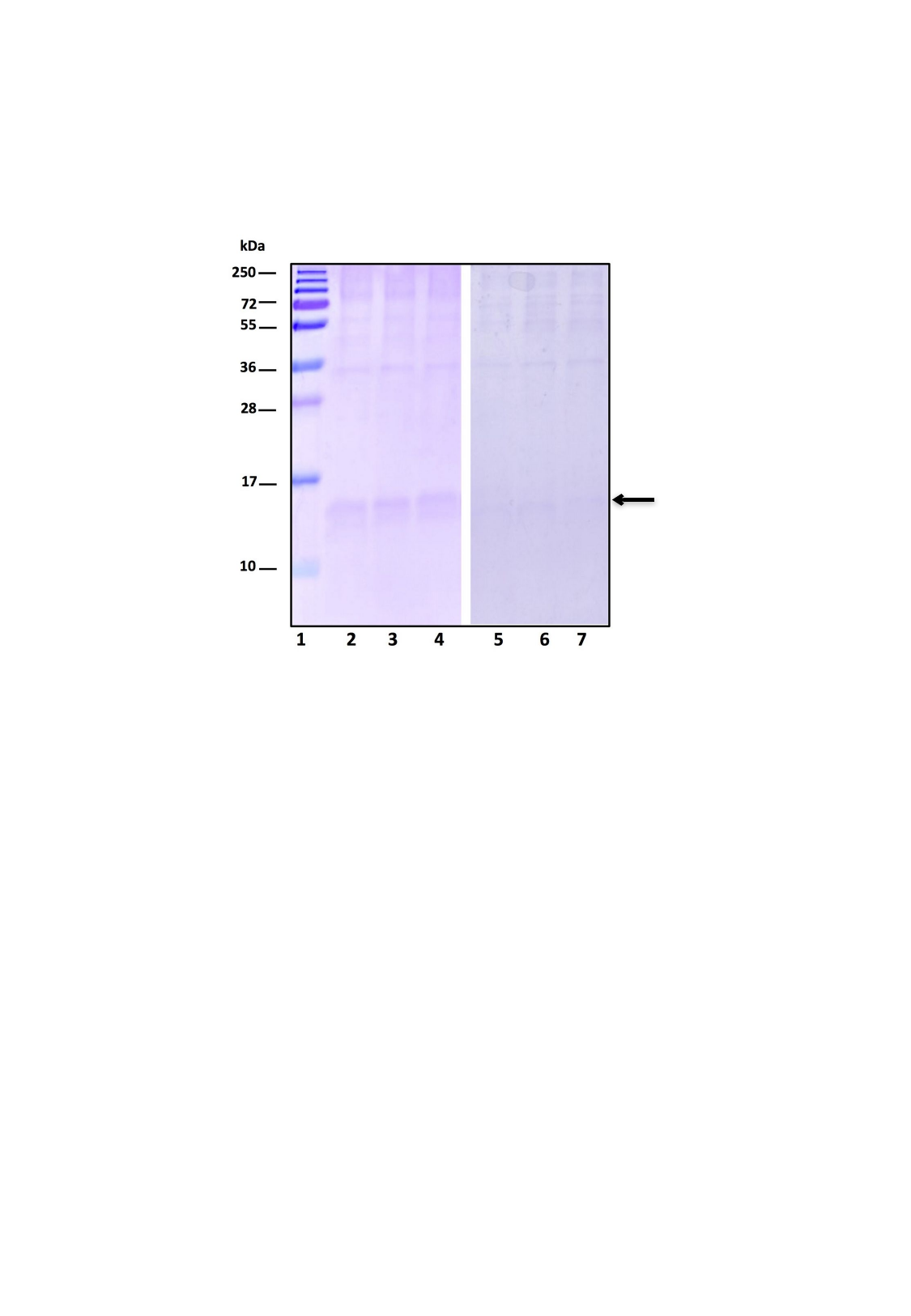

Supplement: Additional file 6: Figure S6. — Evaluating the proteolytic susceptibility of secreted EDIII in culture supernatant. Proteolytic degradation for secreted EDIII was monitored in cell-free culture supernatants to negate the balance between EDIII secretion and degradation. Culture supernatants from P. pastoris expressing EDIII in the absence of CA, were obtained after 6 days of induction at 20° and 30 °C. Retrieved supernatants were further incubated at their respective induction temperatures and samples were collected at 0, 28 and 93 h interval from both. Samples were thereafter separated on a denaturing polyacrylamide gel and visualized by Coomassie stain. No visible degradation was observed indicating negligible proteolysis of secreted EDIII. Lane 1: Prestained protein marker; Lane 2–4: 20 °C; Lane 5–7: 30 °C; Lane 2 & 5: 0 h; Lane 3 & 6: 28 h and Lane 4 & 7: 93 h. (PPTX 567 kb) [file 12896_2016_243_MOESM6_ESM.pptx]

## Slide 1
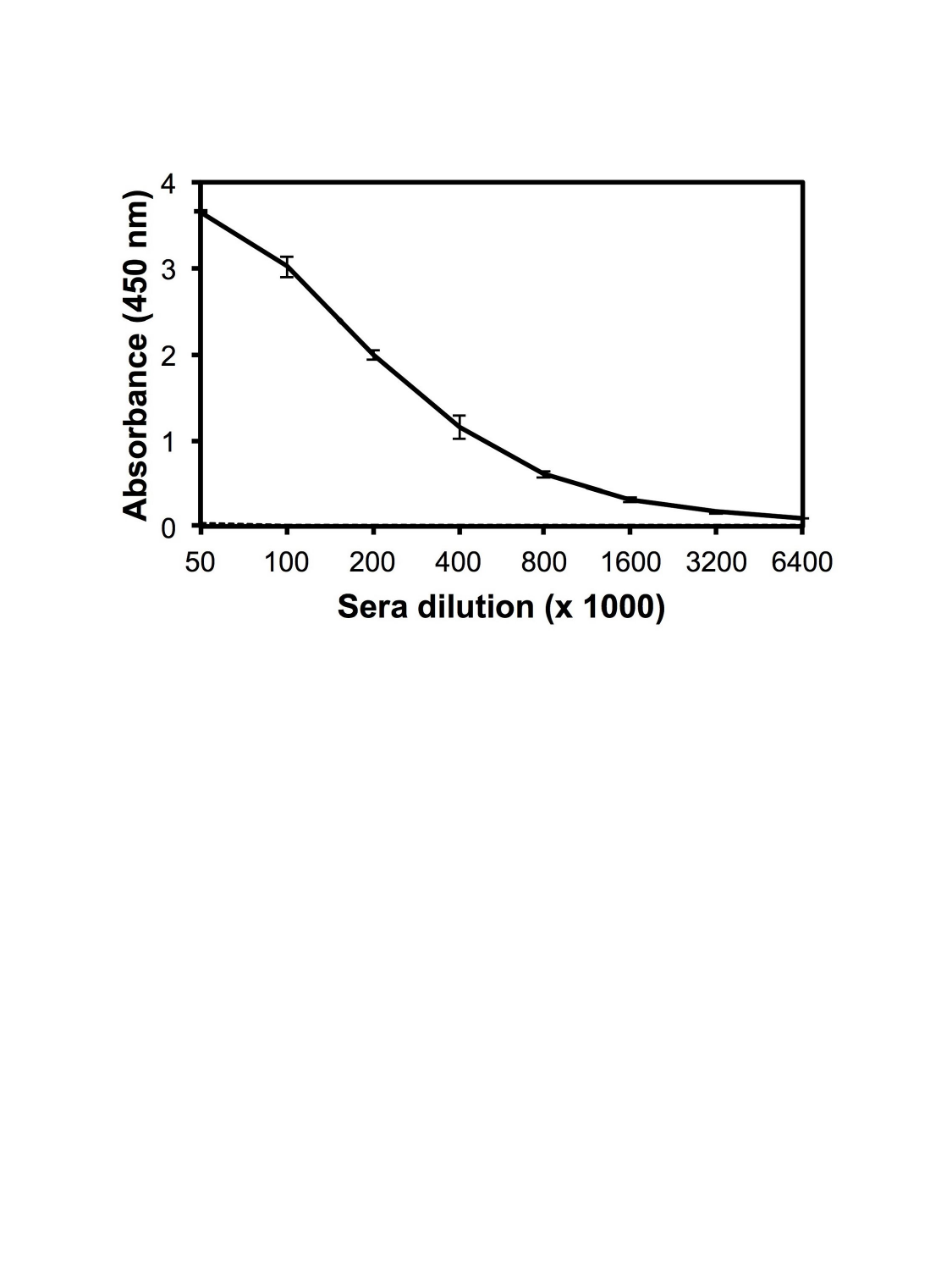

Supplement: Additional file 7: Figure S7. — Evaluation of antibodies elicited by recombinant DENV-3 EDIII using ELISA. Pooled sera from EDIII immunized (solid curve) and mock (dashed curve) BALB/c mice were tested by indirect ELISA using purified EDIII as coating antigen. Goat anti-mouse HRP was used as secondary antibody. Error bars represent the standard deviation calculated from two technical replicate. (PPTX 466 kb) [file 12896_2016_243_MOESM7_ESM.pptx]
