# Supplementary material for: Casamino acids facilitate the secretion of recombinant dengue virus serotype-3 envelope domain III in Pichia pastoris
Source: BMC Biotechnol. 2016 Feb 4;16:12. doi: 10.1186/s12896-016-0243-3 (PMC4743106; doi:10.1186/s12896-016-0243-3)
Supplement: Additional file 8: Table S8. — Absolute values (OD600 and pH) obtained during induction phase. (DOCX 112 kb) [file 12896_2016_243_MOESM8_ESM.docx]

Table S1.

| **Growth at 20°C** | | | | | | | |
| --- | --- | --- | --- | --- | --- | --- | --- |
|  | **Day 0** | **Day 1** | **Day 2** | **Day 3** | **Day 4** | **Day 5** | **Day 6** |
| **0% CA** | 60.0 | 70.2 | 82.1 | 84.3 | 76.5 | 76.2 | 76.3 |
| **0.25% CA** | 60.0 | 78.1 | 84.2 | 88.6 | 84.3 | 84.8 | 84.4 |
| **0.5% CA** | 60.0 | 80.4 | 86.8 | 90.0 | 86.7 | 86.3 | 86.1 |
| **1.0% CA** | 60.0 | 82.4 | 88.3 | 92.4 | 92.2 | 94.6 | 96.8 |
| **1.5% CA** | 60.0 | 84.8 | 90.4 | 94.6 | 94.1 | 96.3 | 100.2 |
| **2.0% CA** | 60.0 | 88.1 | 100.7 | 102.2 | 104.5 | 106.9 | 104.4 |

Table S2.

| **Growth at 30°C** | | | | | | | |
| --- | --- | --- | --- | --- | --- | --- | --- |
|  | **Day 0** | **Day 1** | **Day 2** | **Day 3** | **Day 4** | **Day 5** | **Day 6** |
| **0% CA** | 60.0 | 64.7 | 66.3 | 63.9 | 64.3 | 64.1 | 60.5 |
| **0.25% CA** | 60.0 | 68.6 | 68.2 | 66.2 | 66.5 | 66.3 | 64.6 |
| **0.5% CA** | 60.0 | 74.1 | 72.4 | 72.5 | 70.2 | 70.6 | 68.3 |
| **1.0% CA** | 60.0 | 78.3 | 76.5 | 76.6 | 74.8 | 72.4 | 72.5 |
| **1.5% CA** | 60.0 | 80.4 | 78.2 | 78.3 | 72.4 | 72.3 | 72.2 |
| **2.0% CA** | 60.0 | 80.6 | 78.5 | 76.8 | 66.7 | 64.1 | 62.5 |

Table S3.

| **pH at 20°C** | | | | | | | |
| --- | --- | --- | --- | --- | --- | --- | --- |
|  | **Day 0** | **Day 1** | **Day 2** | **Day 3** | **Day 4** | **Day 5** | **Day 6** |
| **0% CA** | 5.7 | 3.5 | 3.0 | 2.6 | 2.7 | 2.7 | 2.8 |
| **0.25% CA** | 5.7 | 4.3 | 3.2 | 3.1 | 3 | 3 | 3.2 |
| **0.5% CA** | 5.6 | 5.9 | 5.4 | 5.0 | 4.6 | 4.6 | 4.8 |
| **1.0% CA** | 5.6 | 6.4 | 6.3 | 6.3 | 6.1 | 5.9 | 5.9 |
| **1.5% CA** | 5.6 | 6.8 | 6.8 | 6.8 | 6.6 | 6.6 | 6.5 |
| **2.0% CA** | 5.6 | 7.2 | 7.1 | 7.0 | 6.8 | 6.8 | 6.7 |

Table S4.

| **pH at 30°C** | | | | | | | |
| --- | --- | --- | --- | --- | --- | --- | --- |
|  | **Day 0** | **Day 1** | **Day 2** | **Day 3** | **Day 4** | **Day 5** | **Day 6** |
| **0% CA** | 5.7 | 4.9 | 4.9 | 5.0 | 5.6 | 5.8 | 6 |
| **0.25% CA** | 5.7 | 5.9 | 5.8 | 5.9 | 6.0 | 6.3 | 6.4 |
| **0.5% CA** | 5.6 | 6.1 | 6.3 | 6.3 | 6.3 | 6.4 | 6.6 |
| **1.0% CA** | 5.6 | 6.5 | 6.8 | 6.7 | 6.9 | 6.9 | 6.9 |
| **1.5% CA** | 5.6 | 6.9 | 7.1 | 7.2 | 7.3 | 7.3 | 7.3 |
| **2.0% CA** | 5.6 | 7.2 | 7.5 | 7.6 | 7.5 | 7.5 | 7.4 |
