# Supplementary material for: Casamino acids facilitate the secretion of recombinant dengue virus serotype-3 envelope domain III in Pichia pastoris
Source: BMC Biotechnol. 2016 Feb 4;16:12. doi: 10.1186/s12896-016-0243-3 (PMC4743106; doi:10.1186/s12896-016-0243-3)
Supplement: Additional file 9: — Data from another set of independent induction experiment. (PDF 1054 kb) [file 12896_2016_243_MOESM9_ESM.pdf]

Data from another set of independent induction experiment

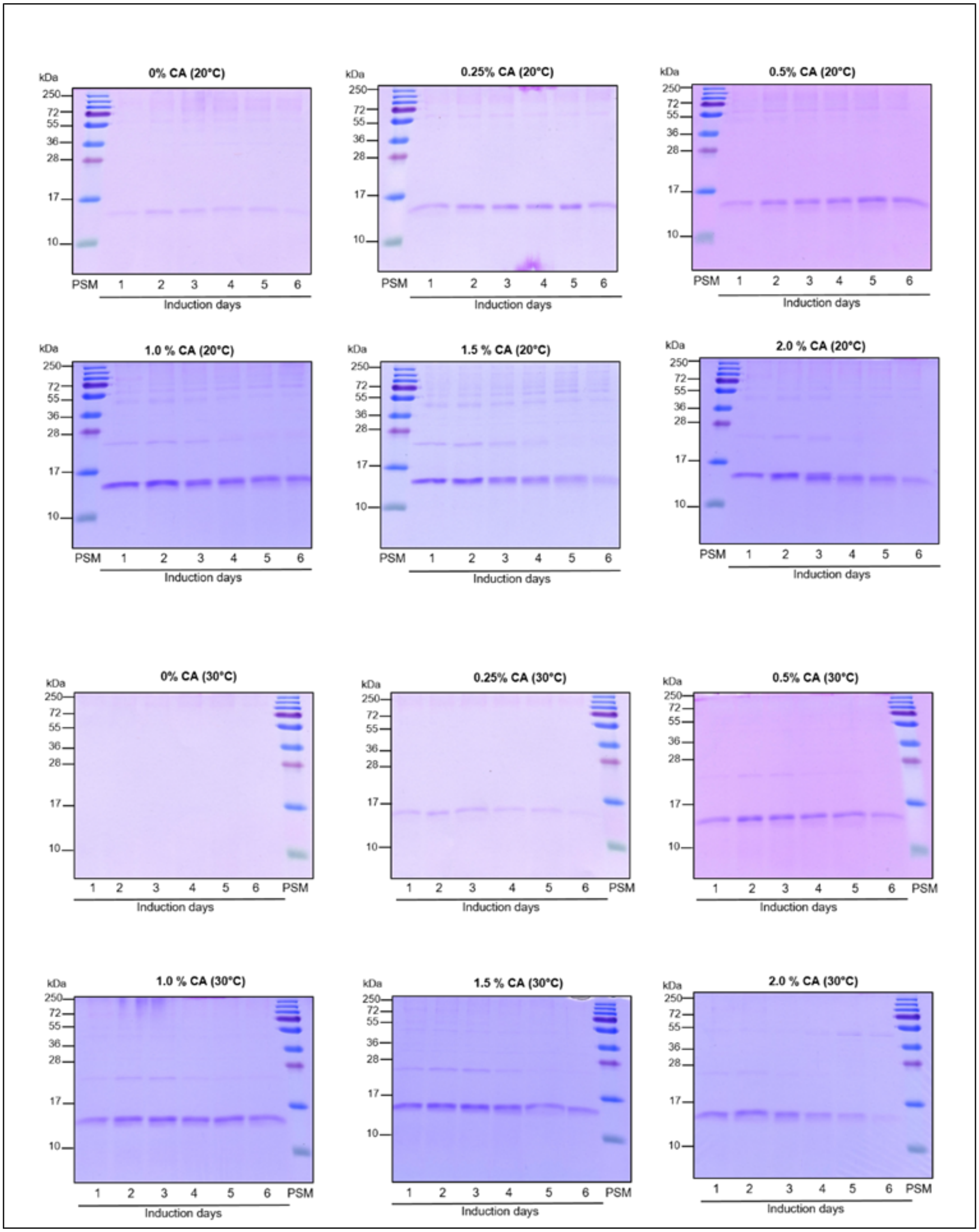

Coomassie stained SDS-PAGE showing secreted EDIII (from independent induction experiment). EDIII levels were evaluated in the supernatant of cultures induced in the absence (CA 0%) or presence of different concentrations of casamino acids (CA 0.25% to 2.0% w/v) supplemented BMM media from day 1 to day 6 at 20°C and 30°C.

Data from another set of independent induction experiment

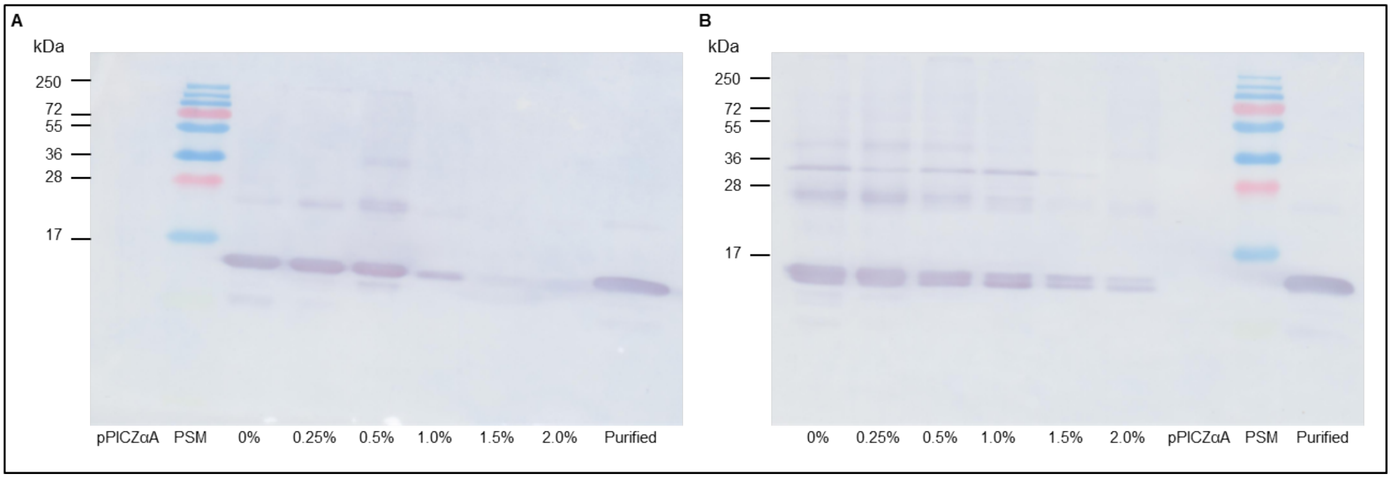

Determination of intracellularly retained EDIII (from independent induction experiment). Western blot of induced *P. pastoris* cell lysate [day 4 harvest with different CA concentration 0.0% to 2.0% (w/v) supplemented BMM media] to determine intracellular EDIII protein at 20°C **(A)** and 30°C **(B)**. pPICZαA is negative control and purified secreted EDIII is positive control.

Data from another set of independent induction experiment

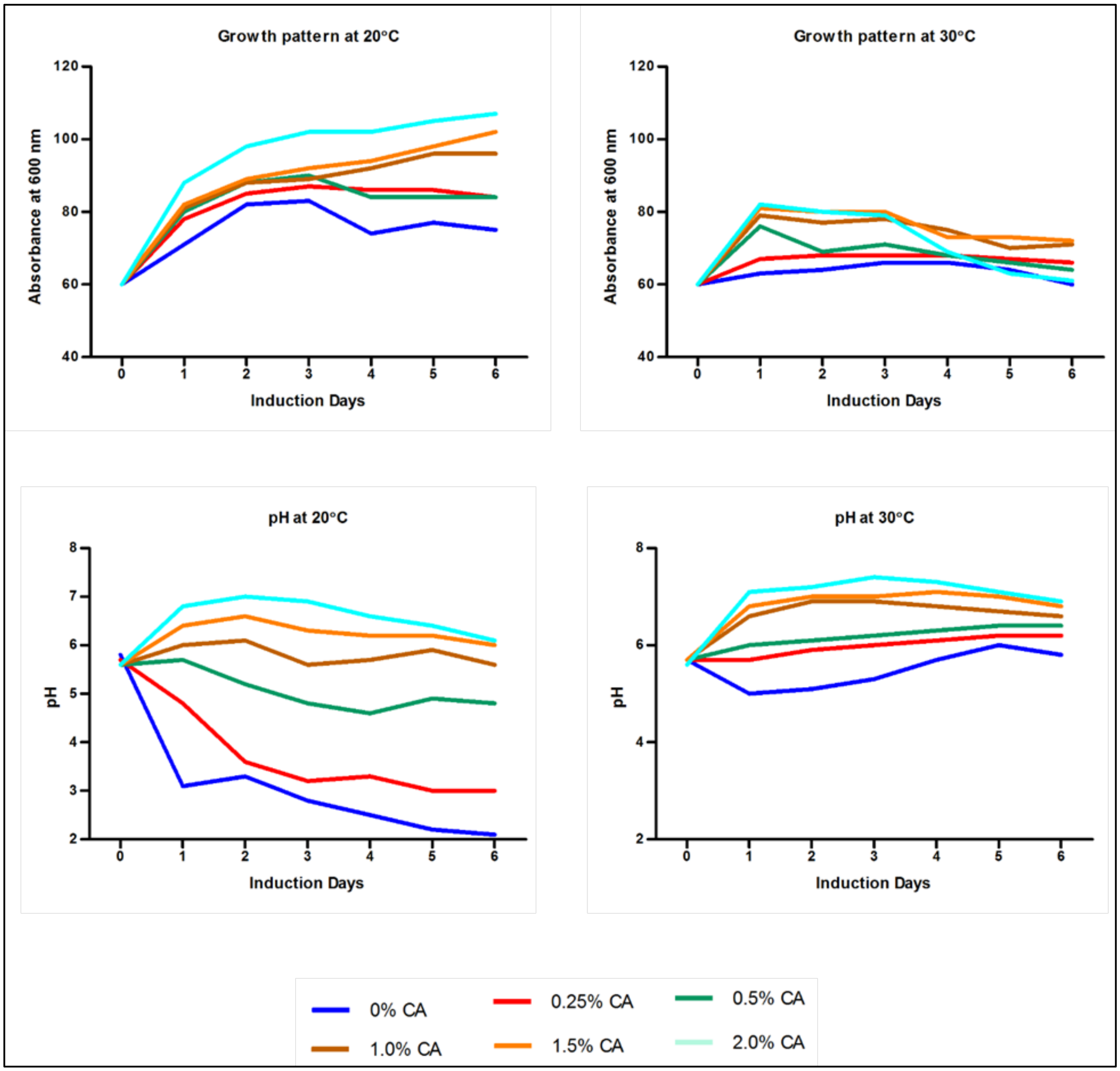

Effect of temperature and CA supplementation on cell growth and culture pH (from independent induction experiment). Cultures induced at 20°C and 30°C were observed for variation in growth profile (top) and pH (bottom) over time (days). Growth profile was determined by measuring the cell density for each time point at 600 nm. Cells were harvested and pH was measured for the retrieved cell-free media.
